# Recent Insights into the Role of Unfolded Protein Response in ER Stress in Health and Disease

**DOI:** 10.3389/fcell.2017.00048

**Published:** 2017-05-10

**Authors:** Dan Lindholm, Laura Korhonen, Ove Eriksson, Sulev Kõks

**Affiliations:** ^1^Medicum, Department of Biochemistry and Developmental Biology, Faculty of Medicine, University of HelsinkiHelsinki, Finland; ^2^Minerva Foundation Institute for Medical ResearchHelsinki, Finland; ^3^Division of Child Psychiatry, Helsinki University Central HospitalHelsinki, Finland; ^4^Department of Pathophysiology, University of TartuTartu, Estonia; ^5^Department of Reproductive Biology, Estonian University of Life SciencesTartu, Estonia

**Keywords:** UPR, ER stress, cell signaling, gene regulation, misfolded protein, human disease

## Abstract

Unfolded stress response (UPR) is a conserved cellular pathway involved in protein quality control to maintain homeostasis under different conditions and disease states characterized by cell stress. Although three general schemes of and genes induced by UPR are rather well-established, open questions remain including the precise role of UPR in human diseases and the interactions between different sensor systems during cell stress signaling. Particularly, the issue how the normally adaptive and pro-survival UPR pathway turns into a deleterious process causing sustained endoplasmic reticulum (ER) stress and cell death requires more studies. UPR is also named a friend with multiple personalities that we need to understand better to fully recognize its role in normal physiology and in disease pathology. UPR interacts with other organelles including mitochondria, and with cell stress signals and degradation pathways such as autophagy and the ubiquitin proteasome system. Here we review current concepts and mechanisms of UPR as studied in different cells and model systems and highlight the relevance of UPR and related stress signals in various human diseases.

Progress made in recent years has greatly increased our understanding about mechanisms of protein homeostasis at the organelle level such as the endoplasmic reticulum (ER) and mitochondria observed during cell stress and in disease. In the ER, the unfolded protein response (UPR) exerts an adaptive function in protein quality control to ensure the proper synthesis, secretion, and correct folding of proteins (Walter and Ron, 2011), whilst mitochondria have their own quality control system known as the mitochondrial unfolded protein response, UPR(mt) (Haynes and Ron, 2010). UPR is activated following cell stress in several human disorders, as illustrated in Figure 1, including neurodegenerative diseases, diabetes and metabolic disorders, atherosclerosis, cancer, as well as renal and lung diseases (Wang and Kaufman, 2012; Hipp et al., 2014). UPR can have contrasting effects in these being either cell protective or cell destructive depending on the strength or duration of the primary insult (Tabas and Ron, 2011; Sano and Reed, 2013). Chronic, sustained UPR usually causes cell death and is described as ER stress but a continuously active UPR is a characteristic of many secretory cells (Moore and Hollien, 2012). UPR signaling plays also a role in development and in normal physiological processes and can influence higher brain functions and neuronal plasticity. In the following, we discuss current concepts and molecular mechanisms underlying UPR and UPR(mt) with a focus on human diseases and the possibilities for novel treatments in protein misfolding diseases.

**Figure 1:**
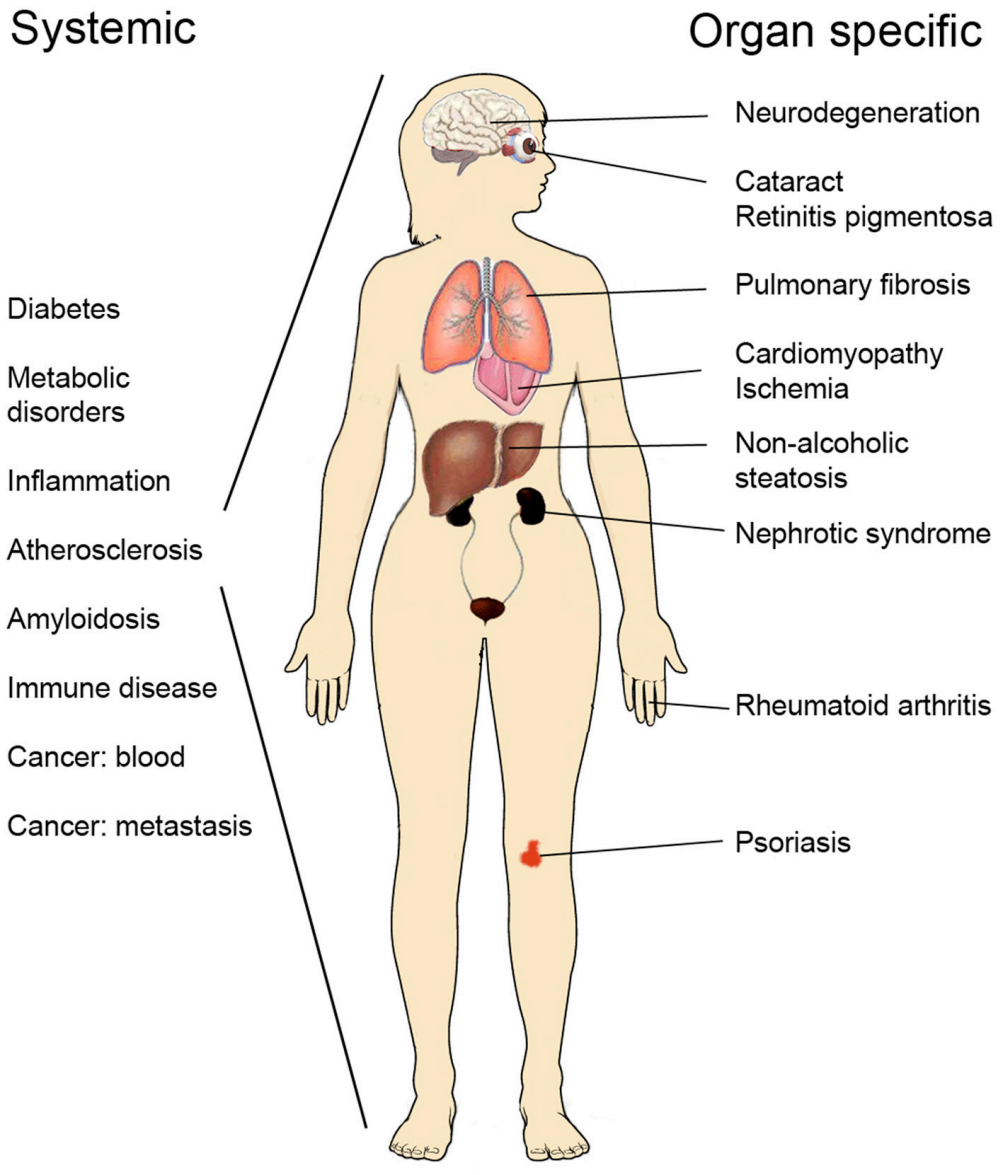
**List of human disorders associated with activation of the unfolded protein response and ER stress**.

## UPR—a friend with multiple personalities

### Sensor proteins for UPR in the ER

During UPR, transmembrane proteins in the ER act as sensors to detect disturbances in protein handling (proteostasis) caused by misfolded/mutant proteins, or by alterations in signaling networks or in the ionic balance such as calcium in the cell. Three major signaling pathways characterize the UPR; the inositol-requiring enzyme 1α (IRE1α), the protein kinase RNA-like endoplasmic reticulum kinase (PERK) and the activating transcription factor 6 (ATF6; Figure 1). These proteins have distinct but overlapping functions in UPR and are structurally composed of specific subdomains. IRE1α has a cytosolic kinase domain that and phosphorylated IRE1α associates with molecules such the c-Jun N-aminoterminal kinase (JNK) involved in cell death regulation (Hetz et al., 2011; Walter and Ron, 2011). IRE1α has also an endoribonuclease activity that cleaves out a 26 bp intron from the pre-mRNA of X-box binding protein-1 (XBP-1) producing an active transcription factor that upregulates expression of genes involved in protein folding (like the ER chaperon GRP78/BiP), in ER-associated protein degradation (ERAD) pathway, and in lipid synthesis. The increased protein folding capacity and degradation of misfolded proteins induced by XBP-1 contribute to restored protein homeostasis and increased cell survival following stress. XBP-1 has also other functions in the cell that are now being explored in more details (see below). In addition, activated IRE1α can cleave certain RNA molecules in a process called IRE1α-dependent decay of mRNAs (RIDD) though the functional significance of this is not fully understood.

Recent studies on the crystal structure of yeast IRE1α have depicted a likely mechanism by which unfolded proteins and peptides can directly bind to and interact with the luminal domain of the molecule. IRE1α then self-associates to produce oligomers that are observed as large foci in the ER using microscopy (Gardner and Walter, 2011). The binding of unfolded peptides to IRE1 has so far been shown mainly using the yeast protein but the mammalian IRE1 shows a rather homologous structure, suggesting similar mode of regulation. This mode of IRE1α regulation by unfolded peptides is at variance with previous thoughts indicating that the binding of BiP is crucial in keeping IRE1α signaling at bay in the ER. However, it is likely that BiP plays a role in fine-tuning of the UPR and overexpression of BiP in different cells reduces ER stress and increases cell survival.

Protein kinase RNA-like endoplasmic reticulum kinase (PERK) is a protein kinase that phosphorylates downstream targets such as the eukaryotic translation initiation factor-2α (eIF2α). This leads to a reduction in general protein synthesis with a decrease in the load of misfolded/mutant proteins in the ER. However, the translational block is not complete and a subset of mRNAs including that encoding the transcription factor ATF4 are still being translated leading to increased expression of among others the growth arrest and DNA damage-inducible 34 (GADD34), the transcription factor C/EBP homologous protein (CHOP) as well as pro-apoptotic BCL-2 family proteins. GADD34, also called PPP1R15A, is a phosphatase for p-IF2α with the ability to restore protein synthesis. The degree of eIF2α phosphorylation is dynamically regulated in the cell and this can be interfered with using specific compounds to control ER stress in various diseases (see below).

The timing of induction of the different UPR pathways and their mutual interactions are important issues and ultimately determine the cell response to stress. Thus, an early and transient increase in IRE1α mediates cell protection, whilst an early-onset increase in PERK-ATF4 causes cell degeneration (Walter et al., 2015). An example of contrasting effects of UPR-controlled signals is also shown by the death receptor 5 (DR5) that is increased by the PERK-CHOP pathway but whose mRNA is cleaved by the endonuclease of IRE1α (Lu et al., 2014). DR5 is important for tumor cell killing and UPR signaling may accordingly influence cancer growth.

In contrast to IRE1α and PERK, ATF6 is first being transported from the ER to the Golgi compartment where it then becomes activated by intramembranous proteolysis mediated by site-1 and site-2 proteases causing the release of the transcriptionally active form of ATF6 (Walter and Ron, 2011). This mode of regulation resembles that of the sterol regulatory element-binding proteins (SREBPs) that are transcription factors for genes involved in lipid and cholesterol metabolism. However, like IRE1α, ATF6 upregulates gene expression of ER chaperons, ERAD components, as well as of XBP-1 (Tabas and Ron, 2011; Sano and Reed, 2013). There are two closely related ATF6 genes, ATF6α and ATF6β and double gene deleted mice are embryonically lethal. ATF6 plays a role in development as studied in the medaka fish (*Oryzias latipes*). ER stress is part of the normal development of the brain and of notochord in medaka, caused by the preponderate synthesis of extracellular matrix (EM) proteins required for organogenesis (Ishikawa et al., 2011). The lack of ATP6 decreased the levels of BiP and other chaperones reducing the folding and transport of EM proteins such as pro-collagen that interferes with early development (Ishikawa et al., 2013). Several proteins are involved in pro-collagen trafficking including the large protein particle (TRAPP) multi-subunit protein complex, and involves large vesicles different from ordinary coat protein complex II (COPII) ones, mediating ER-Golgi transport (Venditti et al., 2012).

### Other ER proteins involved in cell signaling and stress

Members of the astrocyte specifically induced substance (OASIS) protein family are transcription factors that are latent in the ER and activated in a process resembling that of ATF6 (Kondo et al., 2012). These proteins are expressed in a time and cell-specific manner important for their functions. The box B-binding factor-2 human homolog on chromosome 7 (BBF2H7) is present in developing chondrocytes and is activated by ER stress (Izumi et al., 2012). In the nucleus BBF2H7 increases gene expression of proteins involved in the secretion of EM proteins such as collagen. It is considered that BBF2H and ATF6 may act in parallel to enhance cell survival and ensure proper synthesis of EM components during development and after injury.

The nuclear factor erythroid-derived 2-related factor 1 (NRF1) is a transcription factor residing latent in the ER membrane involved in the regulation of expression of antioxidants and ERAD components. NRF1 shares many of its activities with the related factor NRF2, but NRF1 can specifically increase proteins associated with the proteasomes to promote protein degradation. NRF1 can be activated by different factors and events including ER stress (Zhang and Xiang, 2016). Recent studies showed that NRF1 is induced downstream of the target of rapamycin complex-1 (mTORC1) protein that is usually associated with the stimulation of protein synthesis and anabolic processes in the cell. These results indicate that mTORC1 signaling through NRF1 also increases proteasomes enhancing protein turnover, which is equally important for protein homeostasis. The physiological relevance of NRF1 and NRF2 in different cells and human diseases warrants further studies.

Wolframin (WFS1) is a transmembrane ER resident protein encoded by the WFS1 gene that in a mutant form causes Wolfram syndrome 1 (WS1), an autosomal recessive degenerative disease characterized by insulin-dependent diabetes mellitus, optic atrophy, deafness and various neurological symptoms (Hofmann et al., 2003). WFS1 is a component of the UPR and participate in the IRE1 and PERK signaling to maintain ER homeostasis (Fonseca et al., 2005). The promoter region of WFS1 gene has ER stress response element (ERSE) like sequence and WFS1 expression is increased during ER stress by XBP1 and ATF6 (Kakiuchi et al., 2006; Odisho et al., 2015) WFS1 stabilizes the yeast E3 ubiquitin ligase HRD1 (SYVN1 in humans) involved in ERAD and enhances the proteasome-mediated degradation of ATF6α thereby reducing ER stress signaling (Fonseca et al., 2010). Lack of functional WFS1 protein makes cells more vulnerable to different environmental insults (Kõks et al., 2013), and pancreatic β-cells degenerate in WFS1 gene deleted mice subsequent to ER-stress (Ishihara et al., 2004).

## UPR in control of protein secretion

Protein quality control is tightly linked to protein sorting and secretion. Protein export from ER to the Golgi compartment is a highly dynamic process governed by various signals and regulatory molecules (Gomez-Navarro and Miller, 2016). Deregulated trafficking of cell surface proteins occurs in human diseases such as cystic fibrosis (CF) caused by mutations in CF transmembrane conductance regulator gene, CFTR (Schmidt et al., 2016). One major disease-causing mutation in CFTR is called F508del-CFTR and it is found in about two-third of afflicted patients with retention of the misfolded protein in the ER and degraded via the ERAD pathway (Schmidt et al., 2016). Current studies attempt to interfere with the transport of mutant CFR to increase its expression at the cell surface to reverse pathology (Adnan et al., 2016). This is important and may in the future open up new possibilities for treatment of CF and other diseases with protein transport defects. However, a lesson learned from studies of the yeast ATP-binding cassette transporter Yor1p, a homolog of mammalian CFTR, is that the interconnections between protein retention and ERAD of mutant proteins are complex and competing signals control protein trafficking in the ER (Pagant et al., 2007).

Plasma cells are like the insulin producing β-cells are specialized secretory cells with a high biosynthetic capacity. UPR and active IRE1α/XBP-1 signaling is crucial at an early stage during differentiation of the plasma cells from B-lymphocytes. UPR plays an important role during the immune response when large quantities of antibodies are made and secreted by these cells (Cenci et al., 2011). It has been shown that after syntheses are the immunoglobulin heavy chains linked together as larger oligomers in the ER via the C-terminal cysteine. These complexes are subsequently disassembled following UPR to allow for secretion of the immunoglobulin (van Anken et al., 2014). The fine-tuning and function of specific chaperons in this process are important study objectives in the future (Sannino et al., 2014).

UPR plays also a role in secretion of aggregate-prone proteins to the extracellular space. Such proteins are often misfolded and accumulate in tissues in form of soluble aggregates or fibrils as shown in many human amyloidosis diseases (Chen et al., 2015). Activation of UPR can increase the levels of secreted chaperons, as exemplified by the protein ERdj3 (also called B11), and thus influence protein homeostasis also at the cell surface and coordinate it with that inside the cell (Genereux et al., 2015). As shown for mutant transthyretin (TTR) protein, ATF6 signaling is able to counteract amyloid formation and cytotoxicity by reducing secretion of misfolded TTR (Chen J. J. et al., 2014). Recently small molecular compounds were developed that mimic the actions of ATF6 in decreasing amyloidogenic protein accumulation in a process named ER reprogramming by the authors (Plate et al., 2016a). The novel compounds affect secretion of misfolded proteins and are useful tools to study ER stress but may also in the long-run help to design better therapeutics for amyloidosis diseases in human (Eisele et al., 2015).

## Mitochondrial UPR

Like the ER, mitochondria respond to cell stress and have their own protein quality control system known as UPR(mt) (Haynes and Ron, 2010; Jovaisaite et al., 2014). As studied mainly in the worm *Caenorhabditis elegans* the UPR(mt) initiates a cascade of reactions to promote nuclear transcription of mitochondrial chaperones and protective factors for organelle repair and homeostasis. Recently it was shown using myoblasts that the catalytic ATP-dependent Clp protease ATP-binding subunit (ClpX) in the protease complex, ClpXP, degrades misfolded/unfolded or oxidized proteins in the mitochondria during mammalian mitochondrial stress (Al-Furoukh et al., 2015). A novel substrate of the mammalian ClpXP is the matrix protein, nitric oxide-associated protein 1 that is a GTPase involved in the regulation of mitochondrial protein synthesis, cell death and in oxidative respiration (Heidler et al., 2011; Al-Furoukh et al., 2014). Increased oxidative stress is part of human degenerative diseases and involved mitochondria production of reactive oxygen species (ROS). Recent studies showed that the mitochondria inner membrane protease, YME1L is particularly sensitive to ROS resulting in reduced capacity for proteolysis of key mitochondrial proteins during cell stress (Rainbolt et al., 2015). YME1L belongs to the family of ATP-dependent AAA+ quality control proteases including also the AFG3L2 and paraplegin proteins. Mutations in or loss of these protein lead to impaired mitochondria functions and can cause human diseases exemplified by spinocerebellar ataxia type 28 and hereditary spastic paraplegia type 7 (Casari et al., 1998; Di Bella et al., 2010). Alterations in mitochondria are also linked to pathogenesis of different diseases (Nunnari and Suomalainen, 2012). As shown in yeast a decreased mitochondrial protease activity may increase the formation of protein aggregation further worsening cell stress (Erjavec et al., 2013).

In *C. elegans*, the activating transcription factor associated with stress-1 (ATFS-1) is a key factor in the control of UPR(mt) mediating a crosstalk between mitochondria and nucleus. The protein is normally imported into the mitochondria but during cell stress the mitochondrial import machinery is impaired increasing ATFS-1 in the nucleus inducing expression of UPR(mt)-associated genes (Nargund et al., 2012). ATFS-1 is also involved in the innate immunity response and protection against pathogens (Pellegrino et al., 2014). Mutations and deletions in mitochondrial DNA (mtDNA) are associated with UPR(mt) and cellular aging although the situation is complex. Thus, it was shown that UPR(mt) is constitutively activated in a the heteroplasmic *C. elegans* strain that helps to maintain the high amount of mtDNA deletions present (Lin et al., 2016). It remains to be studied whether similar mechanisms prevail in mammalian cells for example during aging. Recently, it was found that the activating transcription factor (ATF5) plays a similar role in mitochondrial stress in mammalian cells as ATFS-1 does in *C. elegans* (Fiorese et al., 2016). The functional relationship of the ATF5-mediated pathway to those of selective mitophagy and to fission/fusion events governing mitochondrial dynamics in mammalian cells and in diseases like Parkinson's disease (PD) requires further studies.

## UPR in neurodegenerative disorders

Neurodegenerative disorders such as Alzheimer's disease (AD), PD, amyotrophic lateral sclerosis (ALS), and Huntington's disease (HD) have all distinct clinical signs band are characterized by the accumulation of misfolded and aggregated proteins (Lindholm et al., 2006; Hetz and Mollereau, 2014). Chronic ER stress contributes to the disease pathology as shown by manipulation of UPR signals using mouse genetics or by specific compounds and drugs. Results indicate that the role of UPR in the different disorders is complex and depends on the timing, strength and nature of the specific pathological insult underlying the disease.

### UPR in ALS, PD, and HD

UPR has been shown to play a dual role in neurodegeneration as the lack of XBP1 increased survival in a ALS mouse model (Hetz et al., 2009). This was unexpected in view of the protective functions associated with IRE1α /XBP1 signaling. Further data showed that XBP1 deficiency increased autophagy through the forkhead box 01 (FOXO1) transcription factor improving the degradation of mutant SOD1 protein (Hetz et al., 2009). In dopaminergic neurons degenerating in PD, XBP1 deletion produce different outcomes depending on the age of the animals and reflecting compensatory changes in UPR-regulated factors. Thus, the lack of XBP1 during development induced a mild ER stress with protection of dopamine neurons against neurotoxicity of 6-hydroxydopamine, whilst silencing XBP1 in adulthood led to chronic ER stress and the loss of dopamine neurons (Valdés et al., 2014). In transgenic mice expressing mutant alfasunyclein as a model for PD, the administration of salubrinal inhibiting the PPP1R15A/GADD34 phosphatase inducing phospho-eIF2α levels was found to be neuroprotective suggesting an involvement of the PERK pathway in dopamine neuron degeneration (Colla et al., 2012).

In an acute neuronal injury, local delivery of XBP1 was beneficial increasing cell survival and the locomotion of animals after a spinal cord lesion (Valenzuela et al., 2012). These findings indicate that manipulation of IRE1α /XBP1 signaling is a possible target for development of therapeutics in different neurological diseases. XBP1 can influence also other functions in neurons than ER stress including learning and memory formation (Martínez et al., 2016). The effect of XBP1 was associated with changes in the expression of brain-derived neurotrophic factor (BDNF) that influences neuronal plasticity and long-term potentiation. Learning and memory are also regulated by the phosphorylation state of eIF2α influenced by PERK as well as other kinases in the neurons (Placzek et al., 2016). The role of UPR in memory-related processed in brain remain an interesting avenue to explore.

In ALS, spinal cord motor neurons and corticospinal motor neurons (CSMNs) in brain cortex undergo cell degeneration by mechanisms only partly understood. Transgenic mice expressing fluorescent labeled ubiquitin C-terminal hydrolase-L1 (UCHL1) in the CSMNs showed that these neurons are particularly sensitive to ER stress and to the lack of UCHL1 the ALS-associated gene, *Alsin* (Jara et al., 2015; Gautam et al., 2016). Cell stress and autophagy contributed to cell degeneration in the CSMNs pointing to novel targets for intervention. Recent data have shown that ALS and frontotemporal dementia share a major genetic risk factor related to the presence of a hexanucleotide repeat expansion in the gene, *C9orf72* (Renton et al., 2011). Studies of CSMNs may give novels insights into the molecular mechanisms underlying the neuronal dysfunctions in these diseases.

One target for intervention in ALS may be PPP1R15A/GADD34 increasing the levels of phospho-eIF2α. Along this line it was shown that the compound guanabenz was able to prolong eIF2α phosphorylation and improve proteostasis in stressed human cells (Tsaytler et al., 2011). However, guanabenz as an adrenergic agonist causes also hypotension and therefore cannot be used in humans. Subsequent screening led to the identification of another molecule, Sephin1 (selective inhibitor of a holophosphatase) that can inhibit the stress-induced phosphatase pathway and restore protein homeostasis (Das et al., 2015). *In vivo* sephin1 was neuroprotective in the mutant SOD1-ALS mouse model, and improved behavior with no obvious adverse effects (Das et al., 2015). Sephin1 also prevented demyelination in Charcot-Marie-Tooth disease model caused by mutant myelin protein zero. Future studies will show whether sephin1 or similar compounds may potentially be useful for treatment of ALS and other protein misfolding disorders in humans.

Studies in models of HD have shown that the lack of XBP1 is neuroprotective by reducing the aggregation of mutant Huntingtin protein (Htt) and increasing cell survival (Vidal et al., 2012). Like in ALS, XBP1 deficiency increased autophagy and upregulated FOXO1 expression in HD (Vidal et al., 2012). As shown recently, the ubiquitin-specific protease 14 (Usp14) plays a role in degradation of mutant Htt by binding IRE1α and by regulating the proteasome activity, adding a further degree of complexity to HD pathophysiology (Hyrskyluoto et al., 2014). The aggregation of mutant Htt may takes place in different neuronal compartments, such as in the nucleus and the cytosol. Recently it was shown that Htt-aggregates are also present at the nuclear membrane interfering with the normal nucleocytoplasmic transport of proteins and RNA (Woerner et al., 2016). Interestingly, expression of TAR DNA binding protein-43 (TDP-43) that is linked to pathophysiology of ALS also affected this transport (Woerner et al., 2016). The significance of nucleocytoplasmic alterations in HD and in other protein misfolding diseases remains to be studied further.

### UPR in AD and prion diseases

AD is associated with accumulation of extracellular plaques consisting of amyloid-β (Aβ) peptides and with intracellular neurofibrillary tangles of hyperphosphorylated tau. The mechanisms for cell toxicity in AD are complex and related to changes in synaptic transmission, an altered calcium homeostasis, increased ER stress and a chronic state of neuroinflammation (Mattsson et al., 2016). Studies made in cell culture and in animal models of AD have shown that the IRE1/XBP1 branch of UPR influences amyloid-β toxicity and is either protective as in Drosophila or deleterious as in *C. elegans* (Cornejo and Hetz, 2013). Moreover, activation of JNK3 by UPR was found to increase production of amyloid-β that aggravated ER stress (Yoon et al., 2012). Inhibition of PERK reducing p-eIF2α levels reversed memory impairments in an AD mouse model (Ma et al., 2013). Together these studies indicate the involvement of specific ER signaling in AD that affects specific neurons and neuronal circuits in various brain regions in a time-dependent manner.

In AD, great efforts have been devoted to study the occurrence and toxicity of different forms of Aβ peptides (Tipping et al., 2015). Amyloid plaques contain also non-protein factors including glycosaminoglycan (GAG) polysaccharides that can increase the rate of Aβ polymerization (Stewart et al., 2016). Solid-state nuclear magnetic resonance spectroscopy has helped to identify the interaction site of Aβ with the polysaccharide heparin as a proxy for GAG (Madine et al., 2012). This information is useful for further structural studies and for design of compounds that may interfere with Aβ fibril formation. Notably, it has been shown that the structure of Aβ fibril in AD brains differ from that *in vitro* and may even be patient-specific for different clinical variants of AD (Lu et al., 2013). More studies into β-amyloid fibrils are therefore warranted both regarding the neuropathology and the possibility for development of novel treatments in AD.

Prion diseases are rare neurodegenerative disorders in humans (such as Creutzfeldt-Jakob disease) and in animals (Bovine Spongiform Encephalopathy) caused by the infectious prion protein (PrP; Colby and Prusiner, 2011). Mutant and misfolded PrP aggregates in brain tissue as amyloid fibrils leading to spongiform changes and loss of brain cells over time. Mounting evidence has shown that the spreading of the disease (propagation) occurs from cell to cell during PrP replication by misfolded PrP causing an abnormal folding of the cellular PrP (Colby and Prusiner, 2011). The self-templating ability of PrP is important for cell toxicity causing an exponential increase in amyloid fibril formation that ultimately causes cell stress and death. In PrP specific prion-like domains are present that influence protein aggregation. Similar domains are also found in other proteins such as in mutant TDP-43 that causes cytoplasmic inclusions in ALS (Smethurst et al., 2016). Prion diseases are important as models to understand the mechanisms of protein aggregation and spreading of infectious agents in neurodegenerative disorders. Recent studies indicate also that a prion-like mechanism can contribute to the cell spreading of α-synuclein in PD (Goedert et al., 2017).

In prion-infected mice, UPR and the PERK pathway was specifically activated with a sustained increase in phospho-eIF2α reduced the synthesis and levels of synaptic proteins causing neuronal death (Moreno et al., 2012). In contrast, inhibition of PERK or an overexpression of the GADD34 phosphatase decreased the p-eIF2α level and improved synaptic functions (Moreno et al., 2013). This lends credence to the view that the modulation of the PERK/p-eIF2α pathway may constitute a drug target for the development of novel therapeutics in prion diseases.

Recent studies using gene expression profiling have revealed changes in prion-infected mouse brain tissue including those in specific microRNAs (Majer and Booth, 2014), and in neuronal-specific genes that influence synaptic and brain functions (Boese et al., 2016). These findings may help to identify important preclinical stages and biomarkers for early diagnoses of prion diseases.

### UPR in brain ischemia and epilepsy

Increased level of the excitatory amino acid glutamate is part of the pathophysiology of brain ischemia and stroke leading to the degeneration and loss of neurons (excitotoxic damage) in the brain. Glutamate acts via different types of glutamate receptors and thereby influences cell signaling cascades and elevates intracellular calcium in neurons. Increased ER stress is linked to brain ischemia (Su and Li, 2016) and to excitotoxic injury as studied by the use of the glutamate receptor agonist, kainic acid (Sokka et al., 2007; Putkonen et al., 2011). Salubrinal that is a specific inhibitor of the eIF2α phosphatase, PP1/GADD34 (Boyce et al., 2005) and was found to protect neurons against excitotoxicity in rat hippocampus and after cerebral ischemia induced by 15 min of brain vessel occlusion in gerbils (Sokka et al., 2007; Anuncibay-Soto et al., 2016).

Epilepsy is characterized by an increased electrical activity in the brain causing repetitive seizures and neuronal dysfunctions. The severity and recurrence of seizures vary between patients and in most cases can be treated with drugs. In chronic temporal lobe epilepsy in humans it was recently shown that ER stress and particularly the IRE1α pathway are becoming activated contributing to brain damage (Liu et al., 2013). This suggests that alleviation of ER stress may be beneficial in reducing neuronal degeneration in epilepsy.

### UPR and neuropsychiatric diseases

Studies of the role of ER stress responses in psychiatric and mental disorders are scarce. Bipolar disorder is characterized mood changes with alternating phases of depression and increase activity (mania). The pathophysiology of BD is complex and the disease is associated with other mental as well as metabolic disorders (Kim et al., 2017). Recent data using patient-derived blood or cell lines s showed that the ER stress response to tunicamycin or thapsigargin is compromised in BD compared with controls (So et al., 2007), suggesting a reduced resistance of cells toward stress in BD. Lithium is a mood stabilizer that is used in the treatment of BD. It was shown that lithium regulates gene network for ER adaptation in BD cells (Breen et al., 2016). Together this suggests that ER stress and associated pathways may prove beneficial targets to consider in treatment of BD (Bengesser et al., 2016) and possibly also in other mood disorders.

Regarding potential ER targets in neuropsychiatric diseases the sigma-1 receptor localized in ER subdomains interacting with mitochondria and is a target for several neuroactive drugs (Hashimoto, 2015). Activation of sigma-1 receptors can afford neuroprotection and modulates ER stress pathways in the brain (Hyrskyluoto et al., 2013; Omi et al., 2014). The precise roles of sigma-1 receptor and other ER resident molecules in neuropsychiatric disease warrant further studies.

## UPR and eye diseases

Photoreceptors in the retina are specialized sensory neurons for detecting light and have a high degree of metabolic activity. Human eye diseases affecting either the rods (responsible for night vision) or cones (responsible for day-light and color vision) are often caused by mutations in specific genes involved in phototransduction or in protein quality control (Chan et al., 2016). Achromatopsia is an eye disease with the prevalence of about 1:30000 that affects primarily cones causing color blindness and reduced vision in afflicted patients. Mutations in cyclic-nucleotide-gated ion channel proteins, governing phototransduction by cGMP, are common causes of congenital forms of achromatopsia. Recently, mutations in ATF6 were found in patients with autosomal recessive achromatopsia, showing that normal ATF6 signaling and BiP levels are crucial for correct protein folding in the photoreceptors (Kohl et al., 2015). The ATF6 mutations are of different types; present either in the luminal part of ATF6 reducing its transport or in the transcriptionally active domain interfering with its activity (Chiang et al., 2017). Future studies will increase our understanding about the role of ATF6 in achromatopsia and other eye diseases.

Retinitis pigmentosa (RF) is a genetically heterogeneous disorder that primarily affects the rods leading to blindness. Mutations in rhodopsin is a common cause of RF and induce an increase in UPR particularly in IRE1α/XBP1 signaling attempting to reduce the amount of mutant rhodopsin in the ER (Chiang et al., 2015). The increase in IRE1α activity precedes the degeneration of the photoreceptors, however how the defects in protein handling ultimately cause cell death is not fully understood (Chiang et al., 2015). Like in other eye diseases, including age-related macular degeneration, autophagy may contribute to the pathology (Kaarniranta et al., 2013).

Cataract is a common eye disorder among elderly people that can cause blindness. The α-crystallins, cryAA, and cryAB, are major molecular chaperons that ensures the solubility of lens proteins throughout life. In cataract, the crystallins are damaged leading to misfolding and aggregation of the proteins into insoluble amyloids impairing vision. As shown recently small molecular compounds can stabilize and reduce aggregation of cryAB in *in vitro* assays (Makley et al., 2015). These novel substances act as pharmacological chaperons and increased lens transparency in a heredity cataract mouse model. Furthermore, they improved lens proteins solubility in human eye samples indicating that they may therefore be considered as a novel treatment for human cataracts (Makley et al., 2015). Chemical chaperons can have beneficial actions in other protein misfolding disorders as well, possibly in conjunction with other treatment strategies.

## UPR and skin diseases

The prevalence of skin disorders is high and these diseases cause much of distress for the patients. In the skin disease vitiligo there is a selective damage of melanocytes (making the pigment) that can occur at any age. It is though that this can be the result of an autoimmune process or caused by reactive oxidative species and subsequent ER stress mediated cell degeneration (Li et al., in press). Cultured melanocytes from vitiligo patients have enlarged ER compared with controls and vitiligo-inducing phenol compounds activate UPR strongly in these cells (Toosi et al., 2012) Genome-wide association studies also support involvement of ER stress genes in vitiligo, and genetic linkage analysis identified an association of XBP1 gene with the development of vitiligo (Birlea et al., 2011).

Psoriasis is the most common chronic inflammatory skin disease. Recently, variations in the autophagy related 16 like-1 gene were found associated with psoriasis vulgaris and palmoplantar pustulosis (Douroudis et al., 2012). The roles of UPR signaling and ER stress in the pathogenesis of psoriasis warrant further studies.

## UPR in metabolic and inflammatory diseases

Many chronic human diseases are characterized by metabolic changes and activation of cell stress signaling that contribute to pathology (Hotamisligil and Davis, 2016). The interactions between metabolism and stress pathways are manifold and involve nutritional signals and hormones such as insulin and controlling anabolic and catabolic pathways. Inflammation is a major component in metabolic diseases and these are consequently named metaflammation disorders (Hotamisligil and Erbay, 2008). Deregulated metabolism may also influence the immune system, and high cholesterol was shown to impair immune cells causing autoimmune diseases (Ito et al., 2016). Increased cell stress and UPR are also frequent in type 2 diabetes, in lipid disorders, in obesity, and in cardiovascular diseases (Hotamisligil and Davis, 2016).

Signaling via Toll-like receptor-2 (TLR2) and TLR4 in macrophages can activate IRE1 and induced XBP1 splicing increasing the production of proinflammatory cytokines contributing the inflammation (Martinon et al., 2010). The IRE1α/XBP1 pathway is activated as shown in cells obtained from synovial fluids of patients with rhemathoid arthritis (Qiu et al., 2013). Treatment with the compound 4μ8C inhibited IRE1α, reduced pro-inflammatory cytokine production and alleviated the joint inflammation in mice with experimental arthritis (Qiu et al., 2013).

### Diabetes and β-cells

Mounting evidence has shown a link between UPR and the development of diabetes with an altered insulin secretion, reduced β-cell viability and increased insulin resistance (IR) in peripheral tissues (Cardozo and Cnop, 2008; Salvadó et al., 2015). Lipotoxicity is a key trigger for β-cell dysfunction and saturated fatty acids are known to induce the PERK and IRE1α pathways (Sommerweiss et al., 2013). Increased insulin synthesis during IR leads to accumulation of misfolded insulin that further activates the UPR (Han et al., 2013). UPR causes accumulation of the human islet amyloid polypeptide, which occurs in 90% of T2D patients (Cadavez et al., 2014). Chaperons ameliorating the ER stress response can increase insulin secretion, pointing to novel therapeutic possibilities in β-cell rescue (Cadavez et al., 2014). The IRE1α/XBP1 branch of UPR plays a critical role in regulation of glucose and lipid metabolism in various diseases (Sha et al., 2011; Jiang et al., 2015). Activated JNK cascade contribute to obesity and insulin resistance by phosphorylation of the insulin receptor substrate, IRS-1 that attenuates insulin signaling (Ozcan et al., 2004).

### Atherosclerosis and heart diseases

In atherosclerosis, macrophages infiltrate the vessel wall and accumulate lipids and lipotoxic substances that sustain the local inflammation. In addition saturated fatty acids such as palmitate can active the IRE1α/XBP1 axis in these cells. The use of the chemical chaperone 4-phenyl butyric acid (4-PBA) was found to mitigate ER stress and reduce the amount of atherosclerotic lesions *in vivo* (Erbay et al., 2009). 4-PBA acts by decreasing the level of the fatty acid-binding protein-4 (aP2) that is crucial for inducing lipotoxic stress in macrophages. Lipid chaperones, acting as fatty acid sensors in these cells, may provide novel targets to consider for treating dyslipidemias (Erbay et al., 2009). In addition, the bioactive lipid palmitoleate also protected against macrophage ER stress and prevented atherosclerosis (Çimen et al., 2016). It remains to be studied whether supplementation with PAO can be of value in treatments of lipid and other metabolic disorders in humans. Recently, the use of the specific IRE1α inhibitor, STF-083010 in mice was shown to reduce atherosclerotic plaque size, lipid-induced inflammation and cytokine production *in vivo* (Tufanli et al., 2017), suggesting that alleviation of ER stress may provide therapeutic benefits also in cardiovascular diseases.

ER stress is also involved in hearth diseases such as myocardial ishemic/infarction and cardiomyopathies. Historically it was shown that heart failure is characterized by morphological changes in the ER, as a hallmark for the ER overload and ongoing stress (Maron et al., 1975). In cellular models, pressure overload induces ER stress and apoptosis in cardiac myocytes (Okada et al., 2004). These finding were confirmed in preclinical experiments with mice. Increased GRP78 expression has been shown in patients with heart failure (Minamino et al., 2010). Myocardial infarction also induces ER stress involving increased oxidative stress reactions. Recent data show a role for the ATF6 pathway in counteracting oxidative stress and counteracting ischemia and reperfusion injury in cardiomyocytes and in mouse hearts *in vivo* (Jin et al., 2017). Activation of XBP1 contributes to tissue adaptation and hypertrophy following heart injury by regulating the expression of the pro-angiogenic factor, vascular endothelial growth factor-A (VEGF-A) *in vivo* (Duan et al., 2016). The regulation of VEGF-A by the IRE1α/XBP1 pathway is probably of general significance in other ischemic disorders as well. Recently, it was reported that sirtuin-1 (SIRT1) counteracts ER stress and reduces the PERK/eIF2α pathway in the injured heart via deacetylation of eIF2α (Prola et al., 2017). This opens up a novel pathway for cardioprotection and for mitigation of ER stress via SIRT1 that itself can be activated by chemical compounds.

In the clinics the use of anticancer drugs can also impinge on the heart to cause dysfunctions in protein quality control events. Cardiotoxicity of such drugs are often linked to the induction of ER stress but also to changes in autophagy and the ubiquitin proteasome system; for a detailed discussion on this see the recent review (Fu et al., 2017).

### Liver metabolism and ER stress

Liver is the central organ in the regulation of metabolism and it ties together different metabolic challenges (lipids, toxins) with the responses in other organs. In many metabolic diseases ER stress and UPR are activated in hepatocytes that eventually can lead to liver damage. UPR in hepatocytes is important to balance fluctuations in blood glucose levels and under diabetic conditions sustained UPR leads to ER stress in the liver (Ji and Kaplowitz, 2006). ER stress itself can induce enzymes involved in gluconeogenesis modulating glucose cycling in the liver (Wang et al., 2006). Altered metabolism in form of diets containing high-carb and high saturated fats can induced accumulation of lipids (steatosis) in the liver characterized by increased ER stress (Sozen and Ozer, 2017). The induction of ER stress and steatosis precedes obesity and changes in insulin action and a sustained ER stress can worsen the metabolic dysregulation through activation of lipidogenic genes. Over-expression of ER chaperone, ORP150 was found to improve insulin resistance and ameliorate glucose tolerance in diabetic animals (Ji and Kaplowitz, 2006). Steatosis and chronic inflammation are also characteristics of alcoholic liver injury. Activation of signaling molecule such as p38 MAPK by inflammatory cytokines can further aggravate lipotoxicity and ER stress and lead to ER/Golgi remodeling (Koeberle et al., 2015). Activation of SREBP signaling by ER stress is responsible for the accumulation of lipids also in alcoholic liver models (Esfandiari et al., 2005). Targeting and relieving ER stress can reverse liver damage and protect also other tissues from harmful effects.

## UPR in lung diseases

ER stress has been shown to play a role in acute lung injuries, in sepsis (Khan et al., 2017), as well in more chronic lung disorders. Increased inflammation accompany lung injuries and treatment with the inhibitor 4-PBA alleviated ER stress and decreased levels of inflammatory cytokines in a mouse model of lung injury and in lung alveolar epithelial cells (Zeng et al., 2017). Lung endothelial cells are also vulnerable to ER stress induced among others by high fat diet causing endothelial cell dysfunctions that were reversed by 4-PBA (Shah et al., 2017). Sepsis is a severe condition with increased ER stress and acute organ failure leading to reduced respiration.

Chronic lung diseases are characterized by increased fibrosis resulting in reduced gas exchange and tissue oxygenation. Chemicals irritations, particle exposition and smoking contribute to the chronic inflammation observed in the lung including a sustained UPR response (Zhang et al., 2017). This in turn leads to the activation of fibroblast, with cellular remodeling and an increased formation of collagen and related molecules in the lung (Lawson et al., 2011). Inflammatory cytokines and growth factors such as transforming growth factor-beta contribute to tissue fibrosis. In the human lung disease, idiopathic pulmonary fibrosis the UPR is activated suggesting novel therapeutic targets (Zhang et al., 2017). Currently novel drug treatments of idiopathic pulmonary fibrosis have been introduced that show therapeutic benefits in the disease (Myllärniemi and Kaarteenaho, 2015; Rochwerg et al., 2016). It remains to be shown whether targeting the UPR pathways may have additional value in the treatments of this or other lung diseases.

Modulation of UPR signaling pathways by different drugs and chemicals has also been used to successfully induce cell death of human lung cancer cells (Zhang et al., 2016; Yang et al., 2017).

## UPR in kidney disorders

In chronic kidney diseases, hypoxia, tissue inflammation, increased oxidative, and ER stress are prevalent findings that together affect the glomeruli and compromise the normal function of the kidney (Maekawa and Inagi, 2017). In particular, podocytes are vulnerable cells and the injury or dysfunctions of these cells can lead to progressive defects in the globular filtration barriers, with proteinuria, and kidney failure as consequences (Maekawa and Inagi, 2017).

Diabetic nephropatia (DN) is characterized by hyperglycemia, increased proteinuria and UPR activation causing podocyte malfunctions and increased cell stress, with advanced glycation end products and free fatty acids present in the kidney (Zhuang and Forbes, 2014). Chronic DN is a major cause of organ failure and death in diabetes calling for preventive measures and treatments. It has recently been shown that tauroursdeoxycholic acid, inhibiting ER stress, counteracted podocyte and glomeruli injury, reduced proteinuria and improved kidney functions in a diabetic mouse model (Fan et al., 2017). In addition, the ER associated protein, reticulon (RTN1A) was identified a critical factor in induction of ER stress in kidney tubular cells (Fan et al., 2017). High expression of RTN1A has also been linked to human DN and other kidney diseases, suggesting a novel target for future alleviations of ER stress in kidney diseases (Fan et al., 2015).

## UPR in aging and trans-cellular communication

Caloric restriction and altered metabolic signaling including insulin/insulin-like growth factor, and mTOR pathways have been linked to longevity in different organisms (Henis-Korenblit et al., 2010; Guarente, 2011). In *C. elegans* the XBP1 pathway regulates resistance to ER stress and lifespan in conjunction with the DAF-16/FOXO signaling (Ben-Zvi et al., 2009). The capacity to mount an UPR response decreases with age (Ben-Zvi et al., 2009). Recent studies have further shown that neuronal overexpression of an active XBP1 increases restores proteostasis not only in neurons but also in distal non-neuronal tissues (Taylor and Dillin, 2013). This trans-cellular communication of UPR signaling requires neuronal activity and may help to coordinate longevity between different tissues in *C. elegans* (Taylor and Dillin, 2013). However, the underlying mechanisms and whether similar processes exist in other organisms remain to be established.

Studies of aging and protein quality control in the yeast, *Saccharomyces cerevisiae* have shown that that damaged and aggregates proteins are segregated between mother and daughter cells so that potentially harmful proteins are eliminated from the progeny. Vacuole fusion in the yeast is important for this process that is controlled by asymmetry-generating genes including the myosin-dependent adaptor protein Vac17 (Hill et al., 2016). Overexpression of Vac17 also reduced the amount of misfolded protein aggregates and prolonged life span in yeast. The tight links shown between the age-dependent increased in protein aggregation and reduced endosome trafficking may have relevance for the aging process in other cells as well. Furthermore, antioxidant enzymes such as the Peroxiredoxins (Prxs), scavenging hydrogen peroxides, modulate aging associated with metabolic signaling. In yeast the anti-aging effect of the antioxidant Tsai involves hyperoxidation of the molecule facilitating the binding of chaperons including the heath shock protein, Hsp70 to misfolded proteins (Hanzén et al., 2016). Prxs and Hsp70 are both elevated in cancer cells and it would be important to know whether a similar mechanism for chaperon regulation occurs in human tumors and other cells.

## UPR and ER stress in cancer

Rapidly growing tumor cells face different types of environmental stressors in form of hypoxia, glucose deprivation, inadequate vascularization, and metabolic challenges. In addition to the external stressors, internal activation of oncogenes, accumulation of somatic mutations, genome instability, changes in chromosome number lead to the additional demands for ER pathways. Tumor cells also adapt to the relative lack of glucose by an increased rate of aerobic glycolysis known as the Warburg effect. To overcome restrains for growth, cancer cells mount an increase in UPR to cope with cell stress and to counteract cell death (Vandewynckel et al., 2013; Kim et al., 2015). Many studies have found sustained and high-level activation of the ER stress genes in variety of solid tumors (Fernandez et al., 2000; Shuda et al., 2003; Moenner et al., 2007). The IRE1α/XBP1 pathway is largely activated in different types of cancer including breast cancer, multiple myeloma, colorectal cancer, and gliomas (Chen X. et al., 2014). IRE1α activation is tumor cells is linked to an increased ER protein folding capacity, ERAD activity, enhanced production of angiogenic factors (VEGF) and restored protein homeostasis. Expression of chaperones renders cancer cells resistant to the pro-apoptotic signals and this correlates to the degree of malignancy of the tumor. The ER chaperones GRP78/BiP and GRP94/HSP90B1 are increased in many different cancers (Moenner et al., 2007). GRP78/BiP plays a role in cancer also by localizing to the cell surface after being released (Tsai et al., 2015). GRP78/BiP can interact with membrane proteins or those in the tumor microenvironment influencing cell signaling cascades, cell proliferation, and immune reactions important for the cancer cell survival (Vandewynckel et al., 2013). In addition, the chaperon GRP170/ HYOU1 has been found to be specifically overexpressed in human breast and thyroid cancers (Kretowski et al., 2013; Zong et al., 2016). Increased PERK activation has also been linked to growth and progression/metastasis of tumors such as colorectal cancer, esophageal, and breast cancer and this can partly be explained by reduced DNA damage an increased angiogenesis (Bobrovnikova-Marjon et al., 2010; Vandewynckel et al., 2013). PERK can also affect the cell cycle and act in a tumor-suppressive manner inducing cell dormancy (Vandewynckel et al., 2013). In view of this and other observation it is not always clear whether sustained ER stress in cancer cells promotes or inhibits tumor growth as this can vary between the different stages of tumor formation (Vandewynckel et al., 2013; Kim et al., 2015).

### Therapeutic possibilities in cancer

As ER stress has obvious role in the survival of cancer cells, IRE1α, and PERK are attractive candidates for drug development (Maly and Papa, 2014; Jiang et al., 2015). Several small molecules have been identified, that are able to inhibit IRE1α. These compounds are shown to have some anti-myeloma activity, but there are serious concerns about the selectivity of these compounds. Mores specific inhibitors, IRE1α kinase inhibiting RNase attenuators (KIRAs) that inhibit IRE1α RNAse, have been developed (Wang et al., 2012). KIRAs are able to inhibit XBP1 RNA cleavage, mRNA decay in ER and IRE1α activity and they are shown to protect against ER stress induced damages (Ghosh et al., 2014).

Potent and selective inhibitors of PERK's kinase domain have also been developed, and one such compound, GSK2656157 revealed promising anti-tumor effects in preclinical models of pancreatic adenocarcinoma and multiple myeloma (Atkins et al., 2013). Main issue with all these inhibitors are their side effects as for examples PERK inhibitors cause pancreatic β-cell loss and diabetes (Oakes, 2017). Therefore, while ER stress is necessary for the cancer progression and spread, the utility of ER stress inhibitors as anti-cancer drugs in the clinics will require careful consideration and critical evaluation of benefits and drawbacks. One possibility for treatment would be to combine UPR inhibitors with those targeting the proteasome as shown recently in case of models of hepatocellular carcinoma (Vandewynckel et al., 2016).

## Conclusions and future prospects

**Figure 2:**
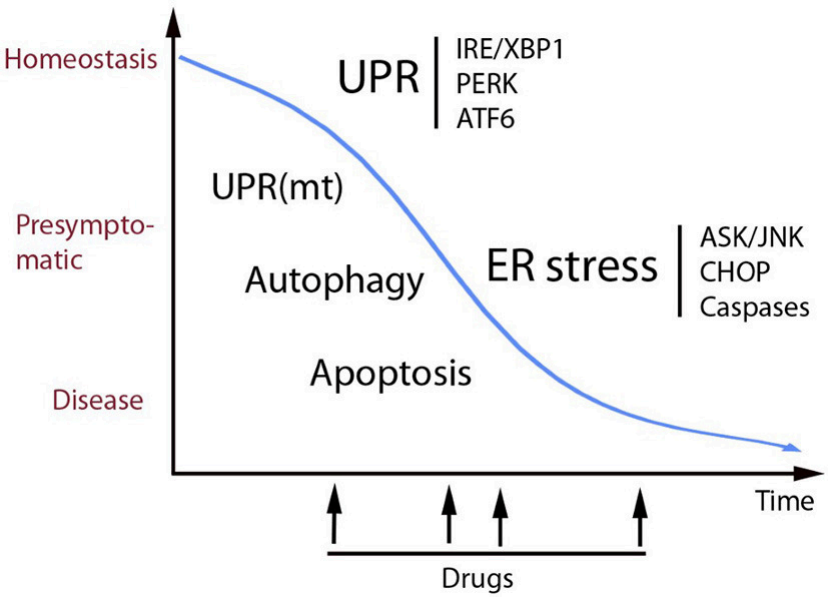
**Schematic view of the role of UPR in cell homeostasis and disease**. UPR is an adaptive pathway in the ER that promotes homeostasis and increases proteostasis (correct folding and secretion of proteins) in the cell. UPR signaling occurs by activation of three transmembrane sensor proteins in the ER (above right) by mechanisms that are described more in the text. Prolonged or heavily sustained UPR can lead to ER stress that can results in full-blow disease. Some of the mechanisms mediating cell degeneration are shown here including the ASK/JNK and CHOP cell death signaling that in many cases involves activation of caspases. Alterations in calcium handling and oxidative stress occurring in ER stress are not depicted here. UPR and ER stress act in concert with other cell stress pathways involving mitochondria and autophagy. Recent studies have identified small molecular compounds that target the UPR signaling pathways, having beneficial actions in various disease models as discussed in the text. In view of the delicate balance between different pathways in homeostasis it is important to know what would be the best timing for drug treatments to alleviate ER stress (see arrows). In addition, chemical chaperons and other therapies are also potentially useful as drugs in different diseases. Increased knowledge about the role of UPR and ER stress and cellular interactions during cell stress can in the long-run lead to novel treatment strategies in various human disorders.

UPR as part of the cell protein quality control system is an important process to optimize cell functions and correct aberrations caused by cell stress. Recent developments in the field have shown that UPR plays a role not only in different diseases but also in physiological processes including the regulation of cell metabolism, inflammation, aging and neuronal plasticity, and cognition. Figure 1 shows some human diseases in which activated UPR/ER stress is thought to play a part. Progress in this field has been facilitated by the development of chemical compounds and inhibitors targeting the UPR signaling pathways with a high degree of specificity (Das et al., 2015; Gallagher et al., 2016; Plate et al., 2016b). Table 1 summarizes small molecules that have been used to target different branches of ER stress in various disorders. The list is only tentative to give a short glimpse into the current state of development and keeping in mind that several experiments are on-going and as such not yet reported. As evident from several studies the timing in the use and the concentration of each compound are important aspects to consider when targeting the different pathways in disease (Figure 2). Mounting evidence also indicates that UPR signaling and ER stress are intimately linked to other cellular pathways such as autophagy and proteasomes involved in protein degradation. In addition, although UPR is associated with ER functions the interactions with organelles such as mitochondria and the UPR(mt) response is important for the final outcome in cell stress. In the future, it will be important to study these interactions further to reveal the precise functions of each signaling pathway and molecules in the cell stress responses in different human pathologies. Given the current pace of research it is foreseen that new discoveries will emerge regarding both the regulatory mechanisms and the possibilities for better treatments of various diseases involving UPR activation and ER stress.

**Table 1:** **Compounds targeting ER stress signaling and used in different experimental settings**.

**Disorder**	**Chemical compound**	**UPR branch/target**	**Effects**	**References**
ALS	Sephin1	Inhibition of stress-induced phosphatase p-eIF2α is increased	Neuroprotection	Das et al., 2015
Excitotoxicity Brain ischemia	Salubrinal	Inhibits GADD34 p-eIF2α increased	Neuroprotection	Sokka et al., 2007; Anuncibay-Soto et al., 2016
PD	Salubrinal	p-eIF2α increased	Neuroprotection in α.synuclein mouse model	Colla et al., 2012
Drug abuse	ISRIB	Increase in the eIF2B guanine nucletide exchange factor	Increase p-eIF2α translation and Induction of LTP by cocaine	Placzek et al., 2016
Prion disease	GSK2606414	PERK inhibitor p-eIF2α reduced	Increase in synaptic proteins and reduced neurodegeneration	Moreno et al., 2013
Inflammation arthritis	4μ8C	IRE1 pathway/ inhibits XBP-1 splicing	reduced cytokines improvement of model of rheumatoid arthritis	Qiu et al., 2013
Multiple myelom	MKC-3946	inhibits XBP-1 splicing	Growth inhibition	Mimura et al., 2012
Pancreatic cancer cell	STF-083010	IRE1 inhibition	Growth retardation	
Tumor cells	Irestin	IRE1-RNase inhibitor, blocks XBP1 effects	Tumor cell survival reduced	
Hepatocellular carcinoma	GSK2656157 or 4μ8C +oprozomib	PERK inhibitor IRE inhibitor Proteasome inhibitor	Antitumor effects	Vandewynckel et al., 2016
Cultured cells	Compound 147	ATF6 activators	Reduced secretion of misfolded protein protein	Plate et al., 2016b
Osteosarcoma cells	Ceapins	ATF6 inhibitor	Sensitizes cells to ER stress	Gallagher et al., 2016

*Summary of small molecules and compounds used to experimentally address ER stress pathways in different disorders. The list gives a glimpse into reported activities in this rapidly progressing field. For more details please see the main text*.

## Author contributions

All authors listed, have made substantial, direct, and intellectual contribution to the work, and approved it for publication.

### Conflict of interest statement

The authors declare that the research was conducted in the absence of any commercial or financial relationships that could be construed as a potential conflict of interest.

## Abbreviations

Aβ: amyloid-β
aP2: fatty acid-binding protein-4
ATF5: activating transcription factor 5
ATF6: activating transcription factor 6
ATFS-1: activating transcription factor associated with stress-1
BBF2H7: box B-binding factor-2 human homolog on chromosome 7
CHOP: C/EBP homologous protein
COPII: coat protein complex II
CF: cystic fibrosis
CFTR: CF transmembrane conductance regulator gene
ClpX: ATP-dependent Clp protease ATP-binding subunit
DR5: death receptor 5
eIF2α: eukaryotic translation initiation factor-2α
ER: endoplasmic reticulum
ERAD: ER-associated protein degradation
FOXO1: forkhead box 01
GADD34: growth arrest and DNA damage-inducible 34
GAG: glycosaminoglycan
Htt: Huntingtin protein
IRE1α: inositol-requiring enzyme 1α
JNK: c-Jun N-terminal kinase
mtDNA: mitochondrial DNA
mTORC1: target of rapamycin complex-1
NRF1: nuclear factor erythroid-derived 2-related factor 1
PERK: Protein kinase RNA-like endoplasmic reticulum kinase
4-PBA: 4-phenyl butyric acid
PrP: prion protein
Prxs: Peroxiredoxins
RF: Retinitis pigmentosa
RIDD: IRE1α-dependent decay of mRNAs
ROS: reactive oxygen species
SREBP: sterol regulatory element-binding protein
TDP-43: TAR DNA binding protein-43
TTR: transthyretin
UCHL1: ubiquitin C-terminal hydrolase-L1
Usp14: ubiquitin-specific protease 14
WFS1: Wolframin
XBP1: X-box binding protein-1.
